# Controlling the Amorphous and Crystalline State of Multinary Alloy Nanoparticles in An Ionic Liquid

**DOI:** 10.3390/nano8110903

**Published:** 2018-11-04

**Authors:** Alba Garzón-Manjón, Hajo Meyer, Dario Grochla, Tobias Löffler, Wolfgang Schuhmann, Alfred Ludwig, Christina Scheu

**Affiliations:** 1 Max-Planck-Institut für Eisenforschung GmbH, Max-Planck-Straße 1, 40237 Düsseldorf, Germany; a.garzon@mpie.de; 2 Werkstoffe der Mikrotechnik, Institut für Werkstoffe, Fakultät für Maschinenbau, Ruhr-Universität Bochum, Universitätsstr.150, D-44801 Bochum, Germany; hajo.meyer@ruhr-uni-bochum.de (H.M.); dario.grochla@rub.de (D.G.); alfred.ludwig@ruhr-uni-bochum.de (A.L.); 3 Analytical Chemistry – Center for Electrochemical Sciences (CES), Faculty of Chemistry and Biochemistry, Ruhr University Bochum, Universitätsstr. 150, D-44780 Bochum, Germany; jan.loeffler@ruhr-uni-bochum.de (T.L.); wolfgang.schuhmann@rub.de (W.S.)

**Keywords:** multinary alloy nanoparticle, ionic liquid, dc sputtering, HiPIMS sputtering, nanoparticle growth, in situ TEM

## Abstract

Controlling the amorphous or crystalline state of multinary Cr-Mn-Fe-Co-Ni alloy nanoparticles with sizes in the range between ~1.7 nm and ~4.8 nm is achieved using three processing routes. Direct current sputtering from an alloy target in the ionic liquid 1-butyl-3-methylimidazolium bis(trifluoromethylsulfonyl)imide leads to amorphous nanoparticles as observed by high-resolution transmission electron microscopy. Crystalline nanoparticles can be achieved in situ in a transmission electron microscope by exposure to an electron beam, ex situ by heating in vacuum, or directly during synthesis by using a high-power impulse magnetron sputtering process. Growth of the nanoparticles with respect to the amorphous particles was observed. Furthermore, the crystal structure can be manipulated by the processing conditions. For example, a body-centered cubic structure is formed during in situ electron beam crystallization while longer ex situ annealing induces a face-centered cubic structure.

## 1. Introduction

Multinary metal nanoparticles (NPs) have attracted increasing interest over the past years for a multitude of applications in optics [1,2], electronics [3], catalysis [4] or other industrial fields [5] due to their unique and tailorable properties [6,7,8]. Synthesis methods for NPs can be divided into bottom-up and top-down processes [9]. The bottom-up processes, using the reduction or decomposition of chemical precursors, allow to achieve a wide variety of NPs. However, the synthesis of multinary NPs is increasingly complicated with the increasing number of constituents and decomposition of by-products, which remain in the suspension and might cause stability issues [10]. As pointed out by Chen et al. [11], there is a large demand for explorative synthesis approaches and the in-depth analysis of multinary NPs as these might possess exceptional properties. 

Physical vapor deposition (PVD), as a top-down technique, allow to prepare NPs by sputtering the elements into ionic liquids (IL) [12]. The ILs act as media for growth and due to their low vapor pressures and melting points, they allow to control the size and shape of the NPs [13,14,15]. Synthesis of metal NPs via sputtering in ionic liquids to produce single element NPs [16,17] or alloy NPs [18,19] and techniques for transfer and accumulation [20,21] have been reported [22]. Co-sputtering from elemental or alloy targets in ILs has almost no limits in the combination of different constituents in multinary NPs and provides a high control in the elemental composition [11,22,23,24]. Still, the formation process in or on the IL itself is not fully understood [13,25]. 

High-power impulse magnetron sputtering (HiPIMS) shows several advantages over conventional direct current (dc) magnetron sputtering, where the sputtered vapor consists mainly of neutral species. HiPIMS provides a high plasma density and a high ionization fraction of the sputtered species by up to 90% [26]. It allows for more control of the thin film growth by controlling the energy and direction of the sputtered species and opens new pathways for the synthesis of multinary NPs. 

In this research work, the formation of quinary alloy Cr-Mn-Fe-Co-Ni NPs by sputtering into IL has been studied to understand in the nanoscale range their excellent catalytic behavior in oxygen reduction reaction (ORR). An intrinsic activity comparable to Pt was recently reported by Löffler et al. [27]. Furthermore, 1-butyl-3-methylimidazolium bis(trifluoromethylsulfonyl)imide IL was used as a stabilizer and suspension media at the same time due to its high stability against decomposition generating stable nanoparticles, as was observed by Meyer et al. [16]. Moreover, it can be utilized in a high vacuum state which has to be applied during sputtering. After conventional dc magnetron sputtering, no crystalline NPs were observed by transmission electron microscopy (TEM) and thus, we classified these as-deposited NPs as amorphous. To obtain crystalline NPs, three processing routes were used: (i) in situ electron beam irradiation, (ii) ex situ heating, and (iii) HiPIMS. 

Aberration-corrected high-resolution TEM (HRTEM) and high-resolution scanning transmission electron microscopy (HRSTEM) coupled with energy-dispersive X-ray spectroscopy (EDS) were used to study the effect of the processing routes on the crystallinity, as well as on the size, shape, and morphology of the multinary NPs.

## 2. Materials and Methods 

### 2.1. Materials

For the synthesis of NPs by sputtering into IL, 1-butyl-3-methylimidazolium bis(trifluoromethylsulfonyl)imide (purity > 99%, [Bmim][(Tf)_2_N]) was purchased from IoLiTec-Ionic Liquids Technologies GmbH (Heilbronn, Germany) (specified impurities were halides (IC) < 100 ppm, and water (KF) < 71 ppm). The IL was stored under an Ar atmosphere and was used with no further purification. 

The custom made alloy sputter target Cr-Mn-Fe-Co-Ni had a purity of 99.95% and a composition of Cr_18_Mn_20_Fe_20_Co_21_Ni_21_ as analyzed by X-ray fluorescence.

### 2.2. Sputter Deposition

The synthesis was performed in a magnetron sputter system (AJA POLARIS-5 chamber from AJA International Inc., Country Way North Scituate, MA, USA) with 1.5” diameter cathodes and a dc power supply (DC-XS 1500). For HIPIMS deposition, a SIPP 2000 USB 10-300-D with an ADL Generator (GS15) from Melec GmbH (Baden-Baden, Germany) was applied. Ar was used as the process gas with a purity of 99.999%.

A multiple cavity holder was applied for sputtering into the IL with a volume of 40 µL per cavity as described elsewhere [16]. With specially designed covers, either 6 or a maximum of 64 cavities are available. For cleaning, the holder was ultrasonicated 20 min each in acetone and isopropanol. The cavities were filled with the IL under an Ar atmosphere and transferred directly into the deposition chamber. After deposition, the holder was removed and transported in an Ar atmosphere and the IL was collected and stored under Ar.

The DC sputter deposition of Cr-Mn-Fe-Co-Ni NPs was performed as follows: the IL [Bmim][(Tf)_2_N] and pieces of a patterned Si/SiO_2_ wafer (2 cm × 3 cm, photolithographically structured with a photoresist lift-off cross pattern for film thickness determination) were stored in the vacuum chamber overnight resulting in a start vacuum of 1.7 × 10^–4^ Pa. After plasma ignition at 1.33 Pa, 20 W and a 2 min precleaning step, the Ar pressure was reduced to 0.5 Pa. A cathode tilt of 12° of the cathode’s normal respective to the normal of the cavity holder was used for the deposition. The deposition was performed at 30 W (312 V, 95 mA) for 2 h. 

HiPIMS sputter deposition of Cr-Mn-Fe-Co-Ni NPs was carried out as follows: The initial steps were the same as described above for dc sputtering with a starting vacuum of 3.0 × 10^–4^ Pa after overnight pumping. To ensure an arc-free and stable HiPIMS plasma, the target ignition was performed gradually. Starting with 400 Hz, 40 W, 30 s (on 40/ off 2560) followed by 200 Hz, 40 W, 30 s (on 40/ off 4960) and finally 100 Hz, 40 W, 30 s (on 40 / off 9960) at an Ar pressure of 2.66 Pa. The HiPIMS deposition was performed with 100 Hz, on 40 / off 9960, 0.5 Pa, average power P_m_ = 40 W (670 V, 30 mA, peak current A_p_ = 27 A, peak current density 2.37Acm^–2^ and a peak power density 1.59 kWcm^–2^) for 3 h providing a film thickness of 270 nm on the patterned reference regions of the wafer.

The multinary dc-as-deposited NPs in the IL were heat treated ex situ under a vacuum (30 Pa) at 100 °C for 2, 5, 10, and 15 h in an oil bath and then cooled down to room temperature in order to verify a possible crystallization. 

### 2.3. Materials Characterization

For TEM investigations, a holey carbon-coated gold grid (Plano GmbH, Wetzlar, Germany) was used, on which a 2.5 µL NP/IL sample was dropped on the coated side and left for adhesion for 2 h. Dried acetonitrile was used to wash the grid dropwise for 1 h under inert conditions. The grid was stored in an Ar atmosphere. Further information can be found elsewhere [16]. 

TEM characterization of multinary NPs was performed in two Titan Themis 60-300 X-FEG (Thermo Fischer Scientific, Eindhoven, Netherlands) equipped either with an image corrector or a probe corrector, both operated at 300 kV. TEM images were recorded on a metal-oxide-semiconductor (CMOS) camera with 4 k × 4 k pixels. High angle annular dark field (HAADF) images (72–352 mrad) and EDS measurements were carried out using STEM with currents of ~90 pA and ~150 pA respectively, a convergence semi-angle of ~23.8 mrad and a beam size of ~0.1 nm. For each sample, up to 200 NPs were investigated.

Beam-induced in situ crystallization of the multinary NPs was achieved at 300 kV with a dose rate ~1.2 × 10^4^ e/nm^2^s, for a total time of 40 min. Crystallization was also possible by using a 200 kV electron beam. To investigate the crystallinity, Fast Fourier transforms (FFT) were calculated from the HRTEM images, always averaging over several NPs. Several NPs contribute to the FFT which was calculated using images of 33.5 × 33.5 nm^2^.

ICP MS analysis was performed with an iCAP RQ ICP MS (Thermo Scientific, Waltham, Massachusetts, USA) employing a TELEDYNE CETAC technologies ASX 560 autosampler. Calibration of elemental concentration was performed with a Q/Qnova calibration solution (Thermo Scientific, Waltham, Massachusetts, USA) in the std mode. A total of 300 µL suspension of sputtered NPs in IL have been dissolved in 69% HNO_3_ (ROTIPURAN Supra of Roth) and diluted with H_2_O to an elemental concentration of approximately 100–200 ppb before transfer to the plasma. Water was purified using an SG purification system yielding a conductivity of 0.055 µS cm^–1^.

## 3. Results

**Figure 1:**
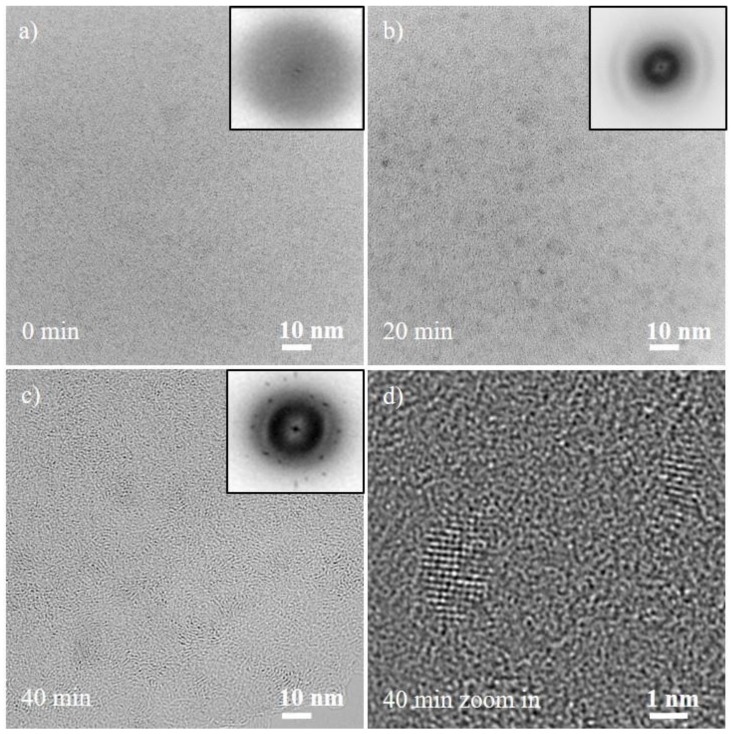
Results of the in situ TEM crystallization experiment for dc-sputtered multinary Cr-Mn-Fe-Co-Ni NPs transferred on a carbon coated gold grid from the IL. (**a**) shows amorphous as-deposited NPs. (**b**) and (**c**) display TEM images and FFT patterns (shown as insets) after 20 and 40 min electron beam illumination and the resulting crystallized NPs. (**d**) HRTEM image of multinary NPs showing lattice fringes. For better visibility, the contrast in the FFT patterns is inverted.

DC sputtering from the Cr-Mn-Fe-Co-Ni target into the IL results in amorphous NPs (see Figure 1a) as no lattice fringes and no reflections in the FFT were observed. Thus, the NPs in the initial state after dc sputtering are referred to as amorphous in the following. For the formation of crystalline multinary NPs, additional processing routes were investigated.

Electron-beam-induced crystallization was carried out to transform the NPs from the amorphous to the crystalline state as shown in Figure 1. After 20 min of electron beam irradiation, the NPs are still amorphous (Figure 1b). Electron beam irradiation of the NPs for more than 40 min leads to a structural transformation and formation of a crystalline state. As a long-range order has been generated, lattice fringes can be observed and reflection spots are present in the FFT (Figure 1c). The atomic arrangement is visible in Figure 1d which shows two NPs at high magnification after the in situ crystallization process.

The phase transformation of the NPs from the amorphous to crystalline state was studied by analyzing the FFT patterns at different stages. At the initial stage (0 min), no reflections are observed. After 20 min, the TEM image was recorded out of focus to get more contrast between the amorphous NPs and the amorphous carbon film to enable the study of the size and the morphology of the NPs in this state. The rings observed in the FFT corresponds to the carbon support. After 40 min of electron beam illumination of the NPs, diffraction spots from the transformation of the NPs to the crystalline state were observed in the FFT (Figure 1c). The reflections can be assigned to the body-centered cubic (bcc) structure: the first ring corresponds to d_1_ = 0.205 nm and is related to the {011} planes and the second ring d_2_ = 0.145 nm to the {002} planes.

The size distribution of the NPs at different stages of the electron-beam assisted crystallization process shows an increase in the diameter in size from 1.7 ± 0.2 nm (amorphous) to 2.6 ± 0.3 nm (crystalline). This increased diameter can be related to the crystallization process and to Ostwald ripening [28]. The histogram of the NPs size distribution is given in the supplementary information (SI) in Figure S1. The NPs are facetted and have a nearly equiaxed shape.

As an alternative approach to electron-beam-induced crystallization of the dc-as-deposited amorphous NPs, ex situ heating was carried out under vacuum (30 Pa) at 100 °C for 2, 5, 10 and 15 h to induce crystallization. The crystal structure was studied using FFT instead of selected area diffraction to avoid the high contribution from the amorphous signal of the carbon-coated TEM grid. 

**Figure 2:**
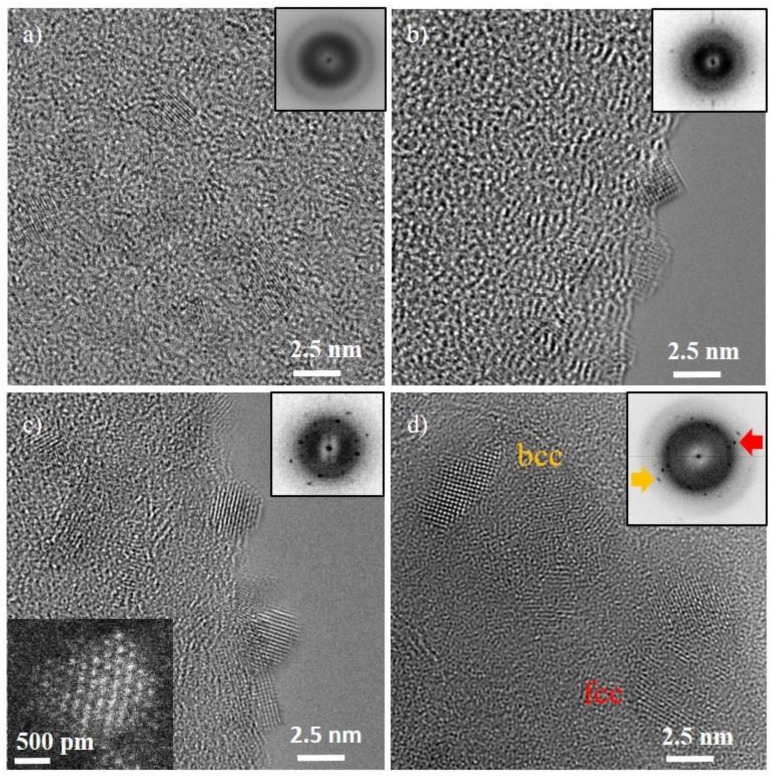
Results of the TEM analysis of Cr-Mn-Fe-Co-Ni NPs after ex situ crystallization at 100°C under vacuum (30 Pa) for (**a**) 2 h, (**b**) 5 h, (**c**) 10 h, and (**d**) 15 h. FFT patterns (insets) show the evolution of the NPs crystallinity. In (a–c) all NPs possess the bcc structure and in (**d**) additional reflections from the fcc phase were found. A HAADF STEM image of one NP is shown in the inset of (**c**). The arrows in (**d**) point to specific reflections of fcc and bcc which can be clearly distinguished.

After two hours of annealing at 100 °C, NPs increase slightly in size (1.9 ± 0.2 nm) and an ordering process starts for the NPs which become crystalline as is visible in their lattice fringes in Figure 2a. After 5 h of annealing, a further increase in order is generated confirmed by the FFT pattern in Figure 2b where more diffraction spots are obtained. The NPs are still 1.9 ± 0.2 nm in diameter. After 10 h, all NPs are crystalline (Figure 2c) and have grown further to 2.4 ± 0.4 nm in diameter. For annealing times up to 10 h, only the bcc crystal structure is observed. The first ring from the FFT patterns corresponds to {011} (d_1_ = 0.205 nm) and the second to {002} (d_2_ = 0.145 nm). The shape of the NP is similar to the in situ case.

After heating the multinary IL NP suspension for 15 h at 100 °C under vacuum, the formation of a phase mixture between bcc and the face-centered cubic (fcc) is observed. The first reflections which are expected for fcc {111} (d_1_ = 0.208 nm) are overlapping with the first ring from bcc {011} (d_1_ = 0.205 nm). The presences of an fcc phase is proven with the second set of reflections which belong to a lattice plane distance d_2_ = 0.180 nm. This value corresponds to the {002} planes of an fcc crystal structure. The NPs have grown further to a diameter of 4.8 ± 0.8 nm as shown in the histogram of the NPs in Figure S2. To understand this change in crystal structure, EDS was performed as discussed below.

**Figure 3:**
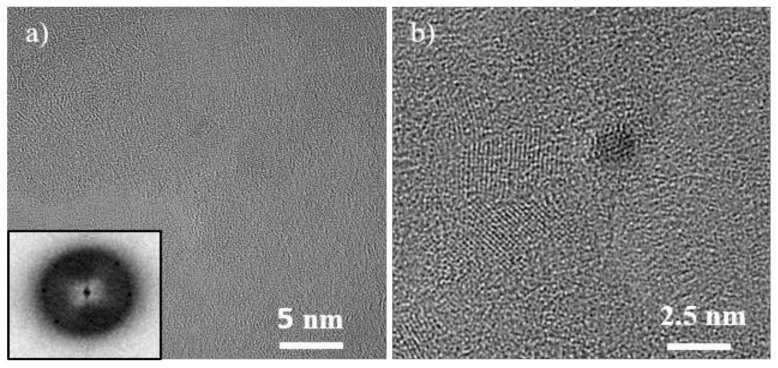
(**a**) TEM image of HiPIMS sputtered Cr-Mn-Fe-Co-Ni NPs transferred on a carbon-coated gold grid from the IL and its corresponding FFT (shown as an inset). (**b**) HRTEM shows bcc lattice planes within the NPs.

With HiPIMS, crystalline NPs are obtained already in the as-deposited state, which also show the biggest diameter of 3.2 ± 0.5 nm. The histogram is provided in the SI (Figure S3). Consistently, they are also facetted and nearly equiaxed. The analysis of the FFT patterns reveals that these NP possess the bcc crystal structure (Figure 3). 

**Figure 4:**
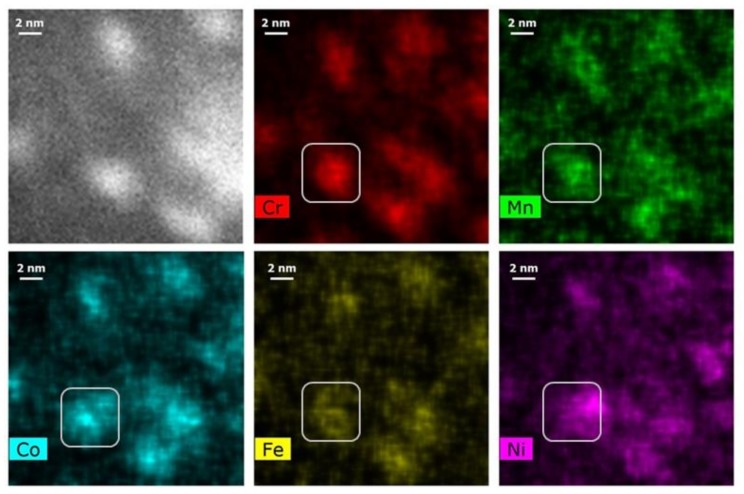
Chemical composition analysis of Cr-Mn-Fe-Co-Ni NPs transferred from the NPs IL suspension onto a carbon-coated gold grid and crystallized with the electron beam. The STEM-EDS maps show the presence and homogeneous distribution of Cr, Mn, Fe, Co, and Ni in each NP. For determination of the atomic composition of single NPs, specific areas were chosen as shown exemplary with a grey rectangle.

EDS analysis in the STEM mode was used to identify and quantify the composition of NPs and to understand the reason for the change from a bcc to fcc structure. The color-coded elemental maps in Figure 4 represent the different chemical elements present in NPs after in situ electron beam crystallization. STEM-EDS requires that the NPs are stable on the grid for a certain time to gain enough counts for a precise single NP quantification. However, NPs were not stable under these conditions. Therefore, maps were recorded with a spatial resolution of ~0.5 nm as exemplarily shown in Figure 4. No individual atomic columns are resolved. In addition, carbon contamination from the support grid and the surrounding IL prevents higher spatial resolution in the EDS maps. Nevertheless, we were able to analyze the changes in the chemical composition of the NPs prepared by the different processes.

The EDS data were analyzed using the Cliff–Lorimer equation [29] and the analysis was done for both, individual NPs and for areas including NPs and their surroundings. The K factors used for the quantification were determined using a reference from bulk Cr-Mn-Fe-Co-Ni material in order to achieve higher accuracy. 

The elemental composition of single multinary NPs with a bcc crystal structure achieved by dc sputtering and electron beam assisted crystallization have according to the STEM-EDS analysis the following range: Cr 33–46 at%, Mn 1–3 at%, Fe 11–15 at%, Co 23–26 at%, and Ni 18–30 at% (see Table 1). After ex situ heating for 15 h at 100 °C, NPs are generated with either a bcc or fcc crystalline structure. Their elemental composition ranges between Cr 17–27 at%, Mn 5–8 at%, Fe 15–17 at%, Co 31–36 at%, and Ni 19–24 at% (see Table 1). The content of Mn, Fe, and Co is similar within the individual NP while the amount of Cr and Ni is differing more strongly. This might be related to the local inhomogeneity of the IL with different tendency to attract or reject these elements. Further studies will clarify this point.

ICP-MS measurements reveal an overall composition in the DC sputtered IL of Cr 31 at%, Mn 4 at%, Fe 11 at%, Co 23 at%, and Ni 31 at% (see Table 1). While the Mn, Fe, and Co contents agree well with the STEM-EDS results, the Cr and Ni content are higher in the ICP-MS measurements. This can be explained as follows: during the ICP-MS analysis, all the elements present in the solution are detected. This means that single clusters from the different elements which are not incorporated in the nanoparticles but located within the IL are also taken into account. 

The NPs synthesized using HiPIMS show an elemental composition between Cr 15–20 at%, Mn 10–19 at%, Fe 19–26 at%, Co 26–31 at% and Ni 13–21 at%. These values obtained by STEM-EDS are also consistent with the ICP-MS of the sample consisting of the NPs and the surrounding IL which still contains some of the sputtered species. The overall composition determined by ICP-MS is Cr 22 at%, Mn 15 at%, Fe 16 at%, Co 22 at% and Ni 25 at%, as shown in Table 1.

## 4. Discussion

In the present study we demonstrated that with sputtering into ILs, multinary NPs with sizes ranging from 2 to 4 nm can be achieved, in both the amorphous or crystalline state. Our multinary NPs prepared using the IL synthesis strategy are much smaller compared to the ones of Chen et al [11]. The small size makes them particularly suitable in different fields such as dye degradation [30], hydrogen evolution [31], methanol oxidation [32] or as ORR catalyst [27]. 

**Figure 5:**
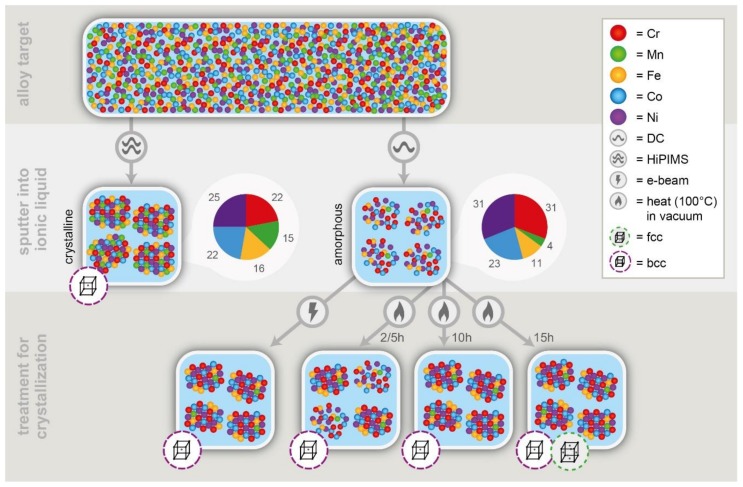
Schematic overview of the different routes used to synthesize amorphous and crystalline multinary NPs. A phase transition from bcc to fcc NPs occurs for ex situ heating at 100 °C within 15h.

The effects of the different sputtering routes (HiPIMS versus DC) and post-deposition treatments are summarized in Figure 5.

Crystalline NPs are obtained by the electron beam and annealing treatment or the HiPIMS process. In our study, the HiPIMS process leads directly to crystalline, multinary NPs. The ionized sputtered species are more energetic than sputtered neutrals and exhibit a broad ion energy distribution up to ~100 eV. Therefore, the nucleation, morphology, and microstructure can be affected [33] and crystalline phases can be formed at lower temperatures [34,35,36,37]. To achieve this, the impinging ion energy and flux have to be tuned by varying the pulse parameters of the HiPIMS discharge.

For HiPIMS and dc sputtering the bcc phase forms, although for the Cantor alloy (equiatomic composition) the fcc crystal structure is expected [38]. STEM-EDS analyses of the dc-as-deposited NPs reveal a low amount of Mn from 1 at% to 3 at%, within the NPs. This might lead to a different crystal structure. The low Mn content is related to the utilized sputter technique. With HiPIMS, the sputtered species have a higher energy and thus, might overcome the surface barrier of the IL easier compared to dc sputtering. Therefore, the composition of the NPs is closer to equiatomic; see Table 1. In the case of ex situ heating at 100 °C for 10 h, the crystallization process is completed and NPs of around 2 to 4 nm in size are produced in accordance to the previously described processes. EDS yields a low Mn concentration of 1–3 at% up to 15 h annealing. After 15 h at 100 °C, the system leads to a phase transition from bcc to fcc, which might be correlated to the incorporation of Mn (up to 8 at%) or temperature. We assume that the changes in composition result from dissolved elements in the solution of the IL after the sputtering process, which are still surrounding the NP and can be incorporated in the crystal lattice during the annealing treatment. 

Although the literature reports the fcc structure for the equiatomic Cr-Mn-Fe-Co-Ni system, we find both bcc and fcc structures for the NPs [39] This might be related to the fact that the crystalline structure can differ for NPs and bulk. Furthermore, the compositional deviation from equiatomic composition (see Table 1) might cause differences in crystal structure. 

## 5. Conclusions

Different pathways for the controlled synthesis of amorphous or crystalline multinary NPs were developed for the Cr-Mn-Fe-Co-Ni system, utilizing combinatorial co sputtering into the ionic liquid [Bmim][(Tf)2N]. Evidently, applying energy to the amorphous NPs leads to crystallization and a larger diameter of the NPs. After in situ electron beam treatment, ex situ annealing up to 15 h or performing the HiPIMS process, a bcc structure was identified. Moreover, after 15 h ex situ annealing, a phase transition from bcc to fcc was observed, which can be related to the change in the elemental composition and to the annealing time, which seems to alter the Mn content of the NPs. We also showed the advantages of HiPIMS sputtering technique compared to dc sputtering which provides crystalline multinary NPs in the as-deposited state and a better control of the chemical composition of the sputtered species in the IL, which makes it a promising synthesis method for multinary NPs in general. The results aim to provide a more in-depth understanding of NPs synthesis methods and how the variation of processing parameters allows for tuning of crystallinity, aspired phase formation and particle size which has important implications for the design and controlled synthesis of target-specific NP.

## Figures and Tables

**Table 1:** Composition range of dc sputtered (as-deposited and 15 h annealed) and HiPIMS sputtered NPs transferred from the NP IL suspension onto a carbon-coated gold grid. Comparison of the STEM-EDS analysis with the overall composition of the sputtered NPs within the IL measured with ICP-MS (*) in at%.

Element	Dc Sputtered			HiPIMS Sputtered	
	As-Deposited in at%	15 h of Annealed in at%	Overall in at%	As-Deposited in at%	Overall in at%
Cr	33–46	17-27	31*	15–20	22*
Mn	1–3	5-8	4*	10–19	15*
Fe	11–15	15-17	11*	19–26	16*
Co	23–26	31-36	23*	26–31	22*
Ni	18–30	19-24	31*	13–21	25*

## Supplementary Materials

The following data are available online at http://www.mdpi.com/2079-4991/8/11/903/s1, Figure S1: Histograms of size distribution of the in-situ TEM crystallization experiment for multinary NPs transferred on a carbon gold grid from the IL a) shows the amorphous state of the NPs in the initial state (1.7 ± 0.2 nm) b) after 40 min electron beam irradiation (2.6 ± 0.3 nm). Figure S2: Histograms of the size distribution of the ex-situ crystallization experiment for multinary NPs at 100 °C under vacuum (30 Pa) transferred on a carbon coated gold grid from the ionic liquid 2 h (1.9 ± 0.2 nm), 10 h (2.4 ± 0.4 nm) and 15 h (4.8 ± 0.8 nm). Figure S3: Histogram of the size distribution of HiPIMS sputtered multinary NPs (3.2 ± 0.5 nm) transferred on a carbon grid from the ionic liquid.
