# Supplementary material for: Controlling the Amorphous and Crystalline State of Multinary Alloy Nanoparticles in An Ionic Liquid
Source: Nanomaterials (Basel). 2018 Nov 4;8(11):903. doi: 10.3390/nano8110903 (PMC6265894; doi:10.3390/nano8110903)
Supplement: Supplementary file 1 [file nanomaterials-08-00903-s001.pdf]

Supporting Information for:

Controlling amorphous and crystalline state of multinary alloy nanoparticles in an ionic liquid

A. Garzón-Manjón[1#], H. Meyer[2#], D. Grochla[2], T. Löffler[3], W. Schuhmann[3], A. Ludwig[2], C. Scheu[1]

1 Max-Planck-Institut für Eisenforschung GmbH, Max-Planck-Straße 1, 40237 Düsseldorf, Germany

2 Werkstoffe der Mikrotechnik, Institut für Werkstoffe, Fakultät für Maschinenbau, Ruhr-Universität Bochum, Universitätsstr.150, D-44801 Bochum, Germany

3 Analytical Chemistry - Center for Electrochemical Sciences (CES), Ruhr-Universität Bochum, Universitätsstr. 150, D-44780, Bochum, Germany

[#]contributed equally

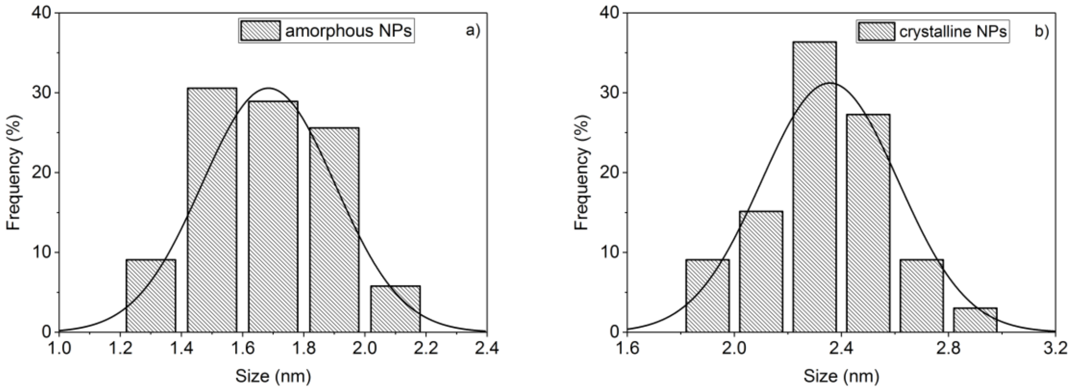

Figure S1. Histograms of the size distribution of the in-situ TEM crystallization experiment for multinary NPs transferred on a carbon coated gold grid from the IL. a) shows the amorphous state of the NPs in the initial state (1.7 ± 0.2 nm). b) after 40 min e-beam irradiation (2.6 ± 0.3 nm).

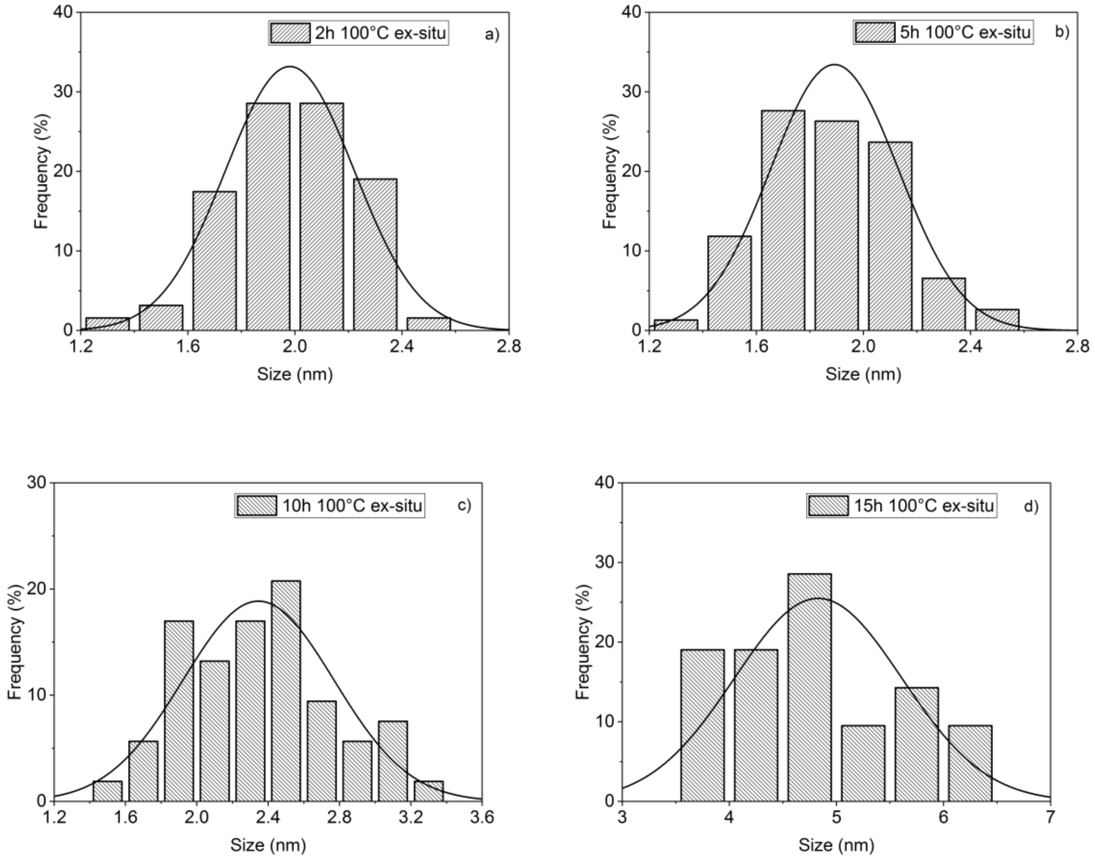

Figure S2. Histograms of the size distribution of the ex-situ crystallization experiment for multinary NPs at 100 °C under vacuum (30 Pa), transferred on a carbon coated gold grid

from the ionic liquid for 2 h (1.9 ± 0.2 nm), 5 h (1.9 ± 0.2 nm), 10 h (2.4 ± 0.4 nm) and 15 h (4.8 ± 0.8 nm).

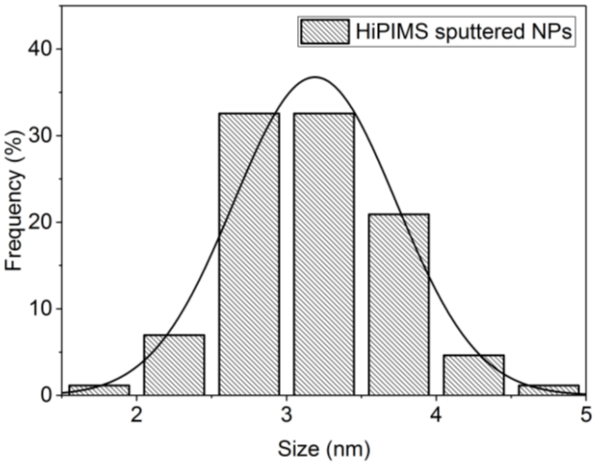

Figure. S3. Histogram of the size distribution of HiPIMS sputtered multinary NPs (3.2 ± 0.5 nm) transferred on a carbon coated gold grid from the ionic liquid.
